# Restrictions on healthcare utilization and psychological distress among patients with diseases potentially vulnerable to COVID-19; the JACSIS 2020 study

**DOI:** 10.1080/21642850.2022.2037429

**Published:** 2022-02-09

**Authors:** Emi Takeuchi, Kota Katanoda, Simone Cheli, Gil Goldzweig, Takahiro Tabuchi

**Affiliations:** a Institute for Cancer Control, National Cancer Center, Tokyo, Japan; b School of Human Health Sciences, University of Florence, Florence, Italy; c School of Behavioral Sciences, The Academic College of Tel Aviv Yafo, Tel Aviv, Israel; d Department of Cancer Epidemiology, Cancer Control Center, Osaka International Cancer Institute, Osaka, Japan

**Keywords:** COVID-19, healthcare, psychological distress, medication, restriction

## Abstract

**Background:**

Patients with comorbidity are at higher risk of deteriorating COVID-19, but they need to access healthcare services regularly for their primary disease. This study aimed to investigate whether patients restricted healthcare utilization due to the COVID-19 pandemic and to understand the relations between restriction of healthcare utilization and psychological distress of patients with a disease potentially vulnerable to COVID-19.

**Methods:**

Participants were a sub-sample of 6,360 individuals suffering from chronic diseases with hypertension, diabetes, respiratory diseases, cerebrovascular disease, and cancer from the nationally representative cross-sectional internet survey data of Japan. Participants reported healthcare utilization during the first state of emergency, as well as psychological distress (K6: The Kessler Psychological Distress Scale 6) in three months after the state of emergency was ended. Multivariate linear regression analyses were performed to examine the correlation between psychological distress and healthcare utilization.

**Results:**

In total, 16% restrained from visiting a hospital as scheduled or canceled a doctor visit. Approximately less than 2% experienced cancellation or delay of hospitalization, treatment, or nursing-care services. After controlling for confounders, multivariate linear regression analysis showed that those who lacked medicines, experienced deteriorated health conditions, and could not visit a hospital during the state of emergency reported severe psychological distress three months later among the patients with all disease types except cancer (*β* = 0.10∼0.25).

**Conclusions:**

The study indicated the restriction in healthcare utilization might be a risk factor for psychological distress among patients. Careful attention to the mental status of patients, especially those who were restricted in healthcare utilization, is necessary.

## Introduction

The novel coronavirus disease 2019 (COVID-19), a highly infectious and potentially fatal disease, has spread rapidly worldwide since a local outbreak in Wuhan, China in December 2019. As of the end of 2021, the number of cases and death is 1.7 million and 18,000 in Japan and 270 million and five million worldwide, respectively (Dong, Du, & Gardner, 2020). This pandemic has generated psychological distress, and especially women, younger adults, individuals with lower SES, living in rural areas, and those with a higher risk of COVID-19 infection severely suffer from psychological distress (Wang, Kala, & Jafar, 2020).

The previous studies have investigated risk factors of severe or fatal outcomes associated with COVID-19. The fact that older people with health conditions are at higher risk from the virus has been widely informed through media. A systematic review study reported obesity, hypertension, diabetes, cardiovascular disease, respiratory disease, cerebrovascular disease, cancer, chronic kidney disease, and liver disease are comorbidities that increase the risk of severe or fatal outcomes associated with COVID-19 (Zhou et al., 2020). In other words, patients with these chronic diseases are at higher risk of deteriorating their health condition when they are infected with COVID-19. Those patients vulnerable to COVID-19 are in the necessity of preventing themselves from the virus with careful attention, but also need to take regular medical treatments for their primary disease. Patients need to balance between both vital needs – COVID-19 restriction and their regular follow-up and treatment.

Some studies reported patients with a chronic disease are likely to feel psychological distress during COVID-19 (Galindo-Vázquez et al., 2020). Some studies suggested that cancer patients reported more preventive behaviors such as washing hands or avoiding public places than healthy controls, and these preventive behaviors were associated with COVID-19 related fear (Musche et al., 2020). These studies indicate that people with a chronic disease might be intensively conscious of protecting themselves from the virus. Patients may be caught in a vicious circle where they restrict themselves because they experience fear of COVID-19 and consequently because of the restriction they feel more fear and depression.

One of the major concerns among people with a chronic disease is reported to be medication deliveries (Lou et al., 2020; Moran, Brooks, & Spoozak, 2020). Decreases in the number of medical consultation, testing, and treatments during COVID-19 has been reported both from the healthcare provider side and patient side (Michalowsky, Hoffmann, Bohlken, & Kostev, 2021; van de Poll-Franse et al., 2021). For example, a study reported 90% of the Spanish patients with chronic obstructive pulmonary disease canceled their medication (Pleguezuelos et al., 2020). Whether cancellation of medication is suggested by patients themselves or health care providers is unknown, but it may lead patients to increase anxiety and fear of deteriorating their health condition. Difficulty to access medication and delay in care were associated with psychological distress among patients with epilepsy and cancer (Frey et al., 2020; Van Hees et al., 2020). Only a few studies reported the association and more studies are necessary to confirm these findings. Also, the level of pandemic and the situation of restriction in healthcare may vary depending on countries, and therefore the investigation in several countries is necessary.

In Japan, a state of emergency for the COVID-19 pandemic was declared for seven metropolitan areas in April 2020 and lifted in May 2020. The government urged residents to stay home during the state of emergency, and schools, theaters, gyms, shops, libraries, museums, nightclubs, and other facilities to close or open in limited hours. Even in this first emergency period, taking medication, commuting to an office, and purchasing groceries were not restricted, and self-restriction on healthcare utilization was not officially expected. However, some patients themselves or medical institutes did restrict going to doctor’s visits or taking treatment because of the state of emergency, and the absence of medical care might have affected their health conditions.

Thus, patients with chronic diseases need to be additionally cautious preventing them from COVID-19 infection. However, more distressed in restricting in healthcare utilization, they may feel more distress in their health problems and adherence. Therefore, this study aimed to investigate whether healthcare utilization was restricted due to the COVID-19 pandemic and how the restriction of healthcare utilization correlated with the mental health of patients potentially vulnerable to COVID-19. The characteristic of chronic diseases varies in type of treatments, severity, and mortality risk, and therefore we investigated the association between healthcare utilization and psychological distress by each disease type.

## Methods

### Study design and participants

The cross-sectional data analysis used the nationwide online survey data from the Japan COVID-19 and Society Internet Survey (JACSIS) (Okubo et al., 2021). The study was approved by the Research Ethics Committee of the Osaka International Cancer Institute, and the National Cancer Center Institutional Review Board. The survey was conducted from August 25 to September 30, 2020, using a Japanese internet survey agency (Rakuten Insight, Inc., Tokyo, Japan https://in.m.aipsurveys.com) registering over 2.2 million panelists. The randomized sampling was conducted by the computer algorithm, and the sample was proportionate to the Japanese general population in age, sex, and living area (i.e. prefecture). A total of 224,389 candidates received an email invitation, and 28,000, which was the targeted final sample size, completed self-reported questionnaires online (response rate = 28,000/224,389 = 12.5%). Participants received the web-based informed consent before answering the questionnaire. To avoid artificial/ unnatural responses, those who chose positive in all of a set of questions for using drugs and having chronic diseases were eliminated from the analysis (*n* = 2,518) as well as those who did not choose the appropriate items for a question ‘please choose the second option from the bottom’. The inclusion criteria of this study were those answered to take treatment for at least one of the following diseases at the time of participating in the survey; hypertension, diabetes, respiratory disease (asthma, pneumonia, and COPD), cardiovascular disease (cardiac infarction, angina, stroke and brain infarction), and cancer. A total of 6,360 participants were included in the analysis.

### Measures

#### Restriction of healthcare utilization

We asked respondents whether they had difficulty in visiting a doctor or taking medical treatments in April and May 2020 which is during a state of emergency for COVID-19 (Appendix 1). The respondents answered eight questions by three choices (yes, no, or none of the above). Four questions regarding the patient's health condition change, the onset of symptoms, maintenance of medication, and hospital visits were asked to all the participants. Those participants with respiratory disease, cardiovascular disease, and cancer who expected to take additional treatments were asked whether they could not hospitalize or take surgery or other treatments due to the COVID-19. Cronbach's alpha is 0.74.

#### Psychological distress

The Kessler Psychological Distress Scale 6 (K6; Kessler et al., 2002), a self-reported measure of psychological distress was used to evaluate psychological distress at the time of participating in the survey (August 2020), which is three months after a state of emergency for COVID-19. That is, there is three months gap between restriction of healthcare utilization and psychological distress was reported. Psychometric properties are presented elsewhere (Furukawa et al., 2008; Kessler et al., 2003). A higher score indicates worse mental health status. The cutoff score for detecting the possibility of severe psychological distress is ≧13 in this study which is consistent with previous studies (Kessler et al., 2003; Min & Lee, 2015; Sakurai, Nishi, Kondo, Yanagida, & Kawakami, 2011).

#### Demographic and other health status data

Demographic data were self-reported, including age, sex, education level, employment status, marital status, comorbidity, history of mental disorder (depression and other mental disorder), chronic pain, and health status. Comorbidity represents those who have more than two types of diseases among the targeted diseases (i.e. hypertension, diabetes, respiratory disease, cardiovascular disease, and cancer). Chronic pain is evaluated by eight questions about the experience of pain on different parts of the body such as the head, chest, and back over the last month. For those who answered to suffer from pain at least one part of their body is regarded as one, while the rest is zero. Cronbach’s alpha is 0.82. Current health status is evaluated by a single question with five Likert scales ranging from one (not good) to five (good). A higher score indicates better health status.

### Statistical analysis

Data analysis was performed using SPSS Statistics, version 24 (IBM, Corp). Missing data were eliminated from the analysis (Appendix 2). To examine restriction in healthcare due to COVID-19 as a risk factor for high distress psychological distress we used multivariate linear regression models with psychological distress (K6) as an outcome measure. Multivariate logistic regression analysis was also conducted with K6 ≧ 13 as a cut-off score and described in Appendices 3 and 4. Demographic factors were entered into each model as potential confounders. Each analysis was conducted by disease types: hypertension, diabetes, respiratory disease, cardiovascular disease. Among the items of restriction in healthcare, the analysis was conducted only when its missing data is less than 60%. The associations between potential risk factors and outcomes were presented as standardized regression coefficients (*β*). The significance level was set at *p* < 0.05 and all tests were two-tailed.

## Results

### Basic characteristics

A total of 6,360 participants was included in this study. As summarized in Table 1, the majority of participants were male (3,914[62%]), full-time workers (2,763[43%]), married (4,464[70%]) and suffered from chronic pain (4,381 [69%]). A total of 939(15%) had been diagnosed with any mental disorder previously.

**Table 1.** Summary of demographics and restriction in healthcare utilization.

	Overall (*N* = 6,360)		Hypertension (*n* = 4,599)		Diabetes (*n* = 1,565)		Respiratory disease (*n* = 1,114)		Cardiovascular disease (*n* = 642)		Cancer (*n* = 455)	
	*N*	%	*N*	%	*N*	%	*N*	%	*N*	%	*N*	%
Age, *M* ± SD	59.99	14.16	62.14	12.39	60.82	13.86	48.76	17.74	56.68	18.38	56.74	18.03
Gender (male)	3,914	61.5	2,938	63.9	1,157	73.9	540	48.5	464	72.3	267	58.7
Education												
high school or below	2,251	35.5	1,643	35.7	574	36.7	355	31.9	241	37.5	137	30.1
college, vocational school or above	4,096	64.5	2,945	64.0	987	63.1	757	68.0	401	62.5	318	69.9
Employment status												
full-time	2,763	43.4	1,945	42.3	733	46.8	538	48.3	295	46.0	190	41.8
part-time	610	9.6	424	9.2	115	7.3	129	11.6	48	7.5	48	10.5
not working	2,987	47.0	2,230	48.5	717	45.8	447	40.1	299	46.6	217	47.7
Marital Status												
married	4,464	70.2	3,354	72.9	1,094	69.9	623	55.9	386	60.1	294	64.6
single	1,062	16.7	636	13.8	287	18.3	340	30.5	151	23.5	106	23.3
divorce	360	5.7	284	6.2	83	5.3	49	4.4	51	7.9	23	5.1
widow	474	7.5	325	7.1	101	6.5	102	9.2	54	8.4	32	7.0
Comorbidity	1,518	23.9	1,315	28.6	969	61.9	455	40.8	505	78.7	289	63.5
History of mental disorder	939	14.8	555	12.1	292	18.7	360	32.3	223	34.7	147	32.3
Pain	4,381	68.9	3,107	67.6	1,032	65.9	867	77.8	464	72.3	317	69.7
Health status^a^, *M* ± SD	3.25	0.99	3.28	0.97	3.09	1.00	3.10	1.06	3.04	1.09	2.97	1.07
Psychological distress (K6 ≧ 13)	479	7.5	263	5.7	120	7.7	187	16.8	87	13.6	54	11.9
Restriction in healthcare utilization												
I got run out of medicines	286	4.5	144	3.1	73	4.7	127	11.4	44	6.9	22	4.8
My condition got deteriorated	253	4.0	141	3.1	87	5.6	103	9.2	46	7.2	29	6.4
I could not visit a hospital as scheduled or canceled a doctor visit	1,025	16.1	673	14.6	252	16.1	256	23.0	126	19.6	66	14.5
I could not visit a hospital for unexpected symptoms or ill condition	380	6.0	227	4.9	88	5.6	128	11.5	57	8.9	28	6.2
I could not get hospitalized	82	1.3	42	0.9	35	2.2	37	3.3	27	4.2	19	4.2
I could not take (or postponed) surgery	92	1.4	58	1.3	34	2.2	40	3.6	30	4.7	21	4.6
I could not take (or postponed) treatment other than surgery	136	2.1	76	1.7	47	3.0	46	4.1	35	5.5	20	4.4
I could not use a nursing-care service	51	0.8	21	0.5	20	1.3	33	3.0	24	3.7	15	3.3

ªHigher score indicates healthier status (range: 1–5).

Table 1 demonstrates that 4,599 (72%) had hypertension, 1,565 (25%) had diabetes, 1,114 (18%) had respiratory disease, 642 (10%) had cardiovascular disease, and 455 (7%) had cancer. Twenty-four percent of the total had multiple diseases.

### Restriction in healthcare utilization

Nearly five percent of the participants experienced running out of medicine, their condition getting deteriorated, and limiting doctor visits for unexpected symptoms or ill conditions. Remarkably, 16% of the participants could not visit a hospital as scheduled or they canceled a doctor visit. Approximately 1–2 percent experienced cancellation or delay of hospitalization, treatment, or nursing-care services.

### The severity of psychological distress and associated demographic factors

Regarding psychological distress, a total of 479 participants (8%) reported severe psychological distress (Table 1). The prevalence was higher in the order from patients with respiratory disease (17%), cardiovascular disease (14%), cancer (12%), diabetes (8%), and hypertension (6%).

As Table 2 demonstrated, younger age (*β* = −0.22∼−0.38, *p* < 0.01), history of mental health (*β* = 0.25∼0.31, *p* < 0.01), chronic pain (*β* = 0.09∼0.13, *p* < 0.01), and worse health condition (*β* = −0.08∼−0.22, *p* < 0.05) were correlated with severe psychological distress which is consistent throughout disease types. Female patients reported severe psychological distress among patients with all the diseases except cancer (*β* = 0.06∼0.09, *p* < 0.05). Among respiratory disease and cardiovascular disease, comorbidity was correlated with severe psychological distress (*β* = 0.07, *p* < 0.01). Similar results were demonstrated with logistic regression analysis in Appendix 3.

**Table 2.** Standardized regression coefficient of psychological distress associated with demographics.

	Hypertension (*n* = 4,599)		Diabetes (*n* = 1,565)		Respiratory disease (*n* = 1,114)		Cardiovascular disease (*n* = 642)		Cancer (*n* = 455)	
	*β*^b^	*p*	*β*^b^	*p*	*β*^b^	*p*	*β*^b^	*p*	*β*^b^	*p*
Age	**−0**.**22**	**<0**.**01**	**−0**.**30**	**<0**.**01**	**−0**.**32**	**<0**.**01**	**−0**.**32**	**<0**.**01**	**−0**.**38**	**<0**.**01**
Gender										
male	0 (reference)		0 (reference)		0 (reference)		0 (reference)		0 (reference)	
female	**0**.**09**	**<0**.**01**	**0**.**05**	**<0**.**05**	**0**.**06**	**<0**.**05**	**0**.**06**	**<0**.**05**	−0.05	0.23
Education										
high school or below	0.01	0.45	0.01	0.82	0.02	0.41	0.02	0.41	−0.01	0.77
college, vocational school or above	0 (reference)		0 (reference)		0 (reference)		0 (reference)		0 (reference)	
Employment status										
full-time or part-time	0.02	0.25	−0.02	0.54	0.02	0.50	0.02	0.50	−0.01	0.82
not working	0 (reference)		0 (reference)		0 (reference)		0 (reference)		0 (reference)	
Marital Status										
married	0 (reference)		0 (reference)		0 (reference)		0 (reference)		0 (reference)	
others	0.02	0.09	−0.01	0.79	0.01	0.73	0.01	0.73	0.05	0.23
Comorbidity	0.00	0.77	0.01	0.51	**0**.**07**	**<0**.**01**	**0**.**07**	**<0**.**01**	0.03	0.45
History of mental disorder	**0**.**25**	**<0**.**01**	**0**.**26**	**<0**.**01**	**0**.**31**	**<0**.**01**	**0**.**31**	**<0**.**01**	**0**.**26**	**<0**.**01**
Pain	**0**.**09**	**<0**.**01**	**0**.**10**	**<0**.**01**	**0**.**12**	**<0**.**01**	**0**.**12**	**<0**.**01**	**0**.**13**	**<0**.**01**
Health statusª	**−0**.**22**	**<0**.**01**	**−0**.**16**	**<0**.**01**	**−0**.**19**	**<0**.**01**	**−0**.**19**	**<0**.**01**	**−0**.**08**	**<0**.**05**

ªHigher score indicates healthier status.

^b^ Adjusted by age, sex, education, employment, marital status, comorbidity, history of mental disorder, pain, and health status; **Bold** *p* < 0.05.

### Restriction in healthcare utilization and its association with psychological distress

Four items of restriction in healthcare utilization were excluded from analysis due to their missing data exceeding 60% of the total (Appendix 2). As summarized in Table 3, after controlling for confounders, multivariate linear regression analysis showed a significant correlation between restriction in healthcare utilization and psychological distress among patients with hypertension, diabetes, respiratory disease, and cardiovascular disease but not cancer. Those who reported ‘I got run out of medicines (*β* = 0.10∼0.17, *p* < 0.05)’, ‘my condition got deteriorated (*β* = 0.10∼0.25, *p* < 0.05)’, ‘I could not visit a hospital as scheduled or canceled a doctor visit (*β* = 0.10∼0.13, *p* < 0.01)’, and ‘I could not visit a hospital for unexpected symptoms or ill condition (*β* = 0.11∼0.19, *p* < 0.01)’ during the state of emergency were more likely to have subsequent psychological distress. The significant correlations were also supported by the logistic regression analysis (Appendix 4).

**Table 3.** Standardized regression coefficient of psychological distress associated with restriction in healthcare.

	Hypertension		Diabetes		Respiratory disease		Cardiovascular disease		Cancer	
	*β*^a^	*p*	*β*^a^	*p*	*β*^a^	*p*	*β*^a^	*p*	*β*^a^	*p*
I got run out of medicines	**0**.**13**	**<**.**01**	**0**.**17**	**<**.**01**	**0**.**12**	**<**.**01**	**0**.**10**	**<**.**05**	0.08	0.13
My condition got deteriorated	**0**.**18**	**<**.**01**	**0**.**25**	**<**.**01**	**0**.**13**	**<**.**01**	**0**.**10**	**<**.**05**	0.03	0.53
I could not visit a hospital as scheduled or canceled a doctor visit	**0**.**11**	**<**.**01**	**0**.**11**	**<**.**01**	**0**.**10**	**<**.**01**	**0**.**13**	**<**.**01**	−0.02	0.72
I could not visit a hospital for unexpected symptoms or ill condition	**0**.**15**	**<**.**01**	**0**.**19**	**<**.**01**	**0**.**14**	**<**.**01**	**0**.**11**	**<**.**01**	0.08	0.12

^a^ Adjusted by age, sex, education, employment, marital status, comorbidity, history of mental disorder, pain, and health status; **Bold** *p* < 0.05.

## Discussion

This study aimed to investigate whether healthcare utilization was restricted due to the COVID-19 pandemic and how the restriction of healthcare utilization correlated with the mental health of patients potentially vulnerable to COVID-19. In total, 16% restrained from visiting a hospital as scheduled or canceled a doctor visit, but the cancellation or delay of hospitalization, treatment, or nursing-care services occurred among only two percent of the participants. After controlling for confounders, multivariate logistic regression analysis showed that the restriction of healthcare utilization correlated with psychological distress, but it varies in the type of disease patients have.

### Possibility of restriction in healthcare use

This study demonstrated whether healthcare utilization is restricted among patients who had a disease that increase the risk of severe or fatal outcomes associated with COVID-19. Although the government did not urge residents to restrict healthcare utilization during the state of emergency in Japan, 16% of the participants reported they could not visit a hospital as scheduled or canceled a doctor visit. Also, the study identified a few percent of the patients experienced the cancellation or delay in hospitalization, surgery, and other treatments. We cannot assume whether patients themselves intended to self-restrict healthcare utilization or cancellation may be the institution side's decision. Hospitalization and treatments might have been canceled because beds, operation rooms, and other medical resources may be assigned to COVID-19 patients on a priority basis and limited among other patients. The study indicated a small but definite number of patients could not get sufficient care as they expected. Further research focusing on this population is necessary to suggest how to ensure adequate utilization of health services and access to medicines in times of this pandemic. Preference in online medical care and remote prescription, or specific needs among this population are some of the topics that should be investigated.

### Prevalence of psychological distress and demographic characteristic associated with psychological distress

As for the prevalence rate of psychological distress, 8% of the participants with disease vulnerable to COVID-19 reported severe psychological distress. The prevalence rate of psychological distress evaluated by K6 was slightly lower than 9–11% among the Japanese general population during COVID-19 (Kikuchi et al., 2020). A previous study showed chronic disease patients reported a higher level of psychological distress compared to healthy individuals (Louvardi, Pelekasis, Chrousos, & Darviri, 2020). In this study, the prevalence of psychological distress or depression varied from 6% to 17% by disease type (e.g. hypertension 6%, diabetes 8%, respiratory disease 17%, cardiovascular disease 14%, and cancer 12%). Previous studies reported 9–24% among cancer patients (Juanjuan et al., 2020; Wang, Duan, et al., 2020), 11% among COPD and asthma patients (Pedrozo-Pupo & Campo-Arias, 2020). The current study showed a similar but a bit lower prevalence of severe psychological distress to previous studies. The Internet survey may include relatively mentally and physically healthier patients compared to other studies most of which recruited participants in medical settings. Another possibility is that some participants reported false diagnoses of diseases and the validity of self-report may be limited. A study reported a positive predictive value of self-reported cancer diagnosis was 60%, which is concluded as an unsatisfied value for study use (Yoshinaga, Sasaki, & Tsugane, 2001). Besides disease type, the result indicated age, gender, marital status, history of mental disorder, chronic pain, and health status were correlated with psychological distress. These correlated factors are known as predictive factors of psychological distress in previous studies.

### Association between restriction in healthcare utilization and psychological distress

The result demonstrated that restriction in healthcare utilization was correlated with severe psychological distress among patients with all disease types except cancer. We assume the sample size of cancer patients was too small to detect the significant correlation between the restriction in healthcare utilization and psychological distress. However, overall, except for cancer patients, this study demonstrated those patients who experienced a lack of medicines and deteriorated conditions, and who could not visit a hospital reported severe psychological distress afterward. Those who have a disease vulnerable to COVID-19 need to pay more careful attention to refrain from going out but also need to take regular medical treatments for their primary disease. Patients may struggle with whether to visit a doctor for medication or should postpone. This conflicted feeling may worsen the patient's mental health. Careful attention to patients’ mental status is necessary as well as maintaining regular care for their primary disease even in the COVID-19 pandemic.

The current study indicated that regardless of mortality risk of disease type, patients with diseases feel severe psychological distress from detachment from medication. We assumed that patients with a disease that have a higher mortality risk like cancer would be more likely to think that restriction of healthcare may lead to death and have more risk of being severely depressed. Opposed to our assumption, the study showed the restriction in healthcare utilization was correlated with psychological distress among patients with diseases that are not a direct cause of death such as hypertension and diabetes. From these findings, we assumed it might be for the first time for patients with lower mortality-risk diseases (i.e. hypertension and diabetes) to witness the risk of death in this pandemic of novel virus disease, and subsequently lead to severe psychological distress. Comparison in disease types, treatment types, frequency of doctor visits, or the period of postponed medication may be necessary as further research with a large sample size to identify patients who psychologically suffer from restriction of healthcare utilization.

### Limitation

A couple of limitations in this study should be raised. First, this is a cross-sectional survey, and therefore the causal relationship should be carefully determined. However, we asked the participants the current status of psychological distress at the time of conducting the survey and restriction in healthcare utilization during the state of emergency, which was three months before the survey, and therefore we expect the possibility of a causal relationship. Second, the participants were asked to report the past status of healthcare utilization, so recall bias is one of the other limitations. Third, the online survey is beneficial in terms of infection prevention and nationwide distribution of surveys in a short period, but the population may be relatively healthier than the targeted population. Last, whether cancellation of medication is suggested by patients themselves or healthcare providers is unknown.

## Conclusion

This study is a first attempt to identify the correlation between restriction in healthcare utilization and psychological distress among patients with chronic disease in Japan. The current study described those patients who experienced the lack of medicines and deteriorated conditions, and who could not visit a hospital reported severe psychological distress afterward. Regardless of the mortality risk of diseases, patients with the diseases vulnerable to COVID-19 may need support to maintain regular care for their primary disease, but also careful attention to their mental status. Screening for psychological distress in regular checkups and referral to psychological specialists may be beneficial as additional care in times of this pandemic.

## Supplementary Material

Supplemental Material

Supplemental Material

## Data Availability

This study was conducted using data collected as a part of JACSIS study. The data are available to any investigators if the request is approved by JACSIS study team.
