# Supplementary material for: Restrictions on healthcare utilization and psychological distress among patients with diseases potentially vulnerable to COVID-19; the JACSIS 2020 study
Source: Health Psychol Behav Med. 2022 Feb 9;10(1):229–40. doi: 10.1080/21642850.2022.2037429 (PMC8843161; doi:10.1080/21642850.2022.2037429)
Supplement: Supplemental Material [file RHPB_A_2037429_SM2608.docx]

Supplementary materials: Japanese translation of the questionnaire

Appendix 1 Translation of Restriction in Healthcare Utilization scale (8 items)

| I got run out of medicines | いつも使っている薬を切らした |
| --- | --- |
| My condition got deteriorated | 持病が悪化した |
| I could not visit a hospital as scheduled or canceled a doctor visit | 予定通りの通院ができなかった（控えた） |
| I could not visit a hospital for unexpected symptoms or ill condition | 予期していなかった症状や病状による通院や受診ができなかった（控えた） |
| I could not get hospitalized | 入院ができなかった（延期になった） |
| I could not take (or postponed) surgery | 治療（手術）ができなかった（延期になった） |
| I could not take (or postponed) treatment other than surgery | 治療（手術以外）ができなかった（延期になった） |
| I could not use a nursing-care service | 自分が利用している介護サービスが受けられなかった |

| Appendix 2 The number and percentage of missing data in Restriction in Healthcare Utilization scale | | |
| --- | --- | --- |
|  | N | % |
| I got run out of medicines | 2187 | 34.4 |
| My condition got deteriorated | 2432 | 38.2 |
| I could not visit a hospital as scheduled or canceled a doctor visit | 2117 | 33.3 |
| I could not visit a hospital for unexpected symptoms or ill condition | 3199 | 50.3 |
| I could not get hospitalized | 4879 | 76.7 |
| I could not take (or postponed) surgery | 4792 | 75.3 |
| I could not take (or postponed) treatment other than surgery | 4372 | 68.7 |
| I could not use a nursing-care service | 5498 | 86.4 |

| Appendix3 Odds ratio for severe psychological distress associated with demographics | | | | | | | | | | | | | | | | | | | | |
| --- | --- | --- | --- | --- | --- | --- | --- | --- | --- | --- | --- | --- | --- | --- | --- | --- | --- | --- | --- | --- |
|  | Hypertension (n=4,599) | | | | Diabetes (n=1,565) | | | | Respiratory disease (n=1,114) | | | | Cardiovascular disease (n=642) | | | | Cancer (n=455) | | | |
|  | N^b^ | %^b^ | Adjusted OR (95%CI)^c^ | | N^b^ | %^b^ | Adjusted OR (95%CI)^c^ | | N^b^ | %^b^ | Adjusted OR (95%CI)^c^ | | N^b^ | %^b^ | Adjusted OR (95%CI)^c^ | | N^b^ | %^b^ | Adjusted OR (95%CI)^c^ | |
| Total | 263 | 5.7 | - | - | 120 | 7.7 | - | - | 187 | 16.8 | - | - | 87 | 13.6 | - | - | 54 | 11.9 | - | - |
| Age, year |  |  |  |  |  |  |  |  |  |  |  |  |  |  |  |  |  |  |  |  |
| <65 | 220 | 4.8 | **3.2** | **(2.1-4.7)** | 109 | 7.0 | **5.1** | **(2.5-10.4)** | 176 | 15.8 | **4.6** | **(2.3-9.1)** | 80 | 12.5 | **4.6** | **(1.8-11.8)** | 49 | 10.8 | **4.6** | **(1.5-13.9)** |
| 65 and older | 43 | 0.9 | 1.0 (reference) | | 11 | 0.7 | 1.0 (reference) | | 11 | 1.0 | 1.0 (reference) | | 7 | 1.1 | 1.0 (reference) | | 5 | 1.1 | 1.0 (reference) | |
| Gender |  |  |  |  |  |  |  |  |  |  |  |  |  |  |  |  |  |  |  |  |
| male | 155 | 3.4 | 1.0 (reference) | | 82 | 5.2 | 1.0 (reference) | | 88 | 7.9 | 1.0 (reference) | | 65 | 10.1 | 1.0 (reference) | | 36 | 7.9 | 1.0 (reference) | |
| female | 108 | 2.3 | **1.4** | **(1.0-1.8)** | 38 | 2.4 | 1.2 | (0.7-1.8) | 99 | 8.9 | 1.3 | (0.9-1.9) | 22 | 3.4 | 0.7 | (0.4-1.3) | 18 | 4.0 | 0.7 | (0.4-1.4) |
| Education |  |  |  |  |  |  |  |  |  |  |  |  |  |  |  |  |  |  |  |  |
| high school or below | 89 | 1.9 | 1.1 | (0.8-1.5) | 40 | 2.6 | 1.2 | (0.8-1.8) | 58 | 5.2 | 1.2 | (0.8-1.8) | 24 | 3.7 | 1.0 | (0.5-1.7) | 10 | 2.2 | 0.7 | (0.3-1.6) |
| college or above | 172 | 3.7 | 1.0 (reference) | | 79 | 5.0 | 1.0 (reference) | | 129 | 11.6 | 1.0 (reference) | | 63 | 9.8 | 1.0 (reference) | | 44 | 9.7 | 1.0 (reference) | |
| Employment status |  |  |  |  |  |  |  |  |  |  |  |  |  |  |  |  |  |  |  |  |
| full-time | 151 | 3.3 | 1.4 | (1.0-2.0) | 80 | 5.1 | 1.5 | (0.9-2.7) | 111 | 10.0 | 1.2 | (0.8-1.8) | 57 | 8.9 | 0.9 | (0.5-1.7) | 37 | 8.1 | 1.3 | (0.6-2.9) |
| part-time | 35 | 0.8 | 1.4 | (0.9-2.3) | 11 | 0.7 | 1.2 | (0.5-2.7) | 19 | 1.7 | 0.7 | (0.4-1.4) | 5 | 0.8 | 0.5 | (0.2-1.4) | 5 | 1.1 | 1.1 | (0.3-3.6) |
| not working | 77 | 1.7 | 1.0 (reference) | | 29 | 1.9 | 1.0 (reference) | | 57 | 5.1 | 1.0 (reference) | | 25 | 3.9 | 1.0 (reference) | | 12 | 2.6 | 1.0 (reference) | |
| Marital Status |  |  |  |  |  |  |  |  |  |  |  |  |  |  |  |  |  |  |  |  |
| married | 150 | 3.3 | 1.0 (reference) | | 62 | 4.0 | 1.0 (reference) | | 80 | 7.2 | 1.0 (reference) | | 32 | 5.0 | 1.0 (reference) | | 21 | 4.6 | 1.0 (reference) | |
| single | 79 | 1.7 | 1.2 | (0.9-1.7) | 42 | 2.7 | 1.0 | (0.6-1.6) | 82 | 7.4 | 1.2 | (0.8-1.7) | 38 | 5.9 | 1.1 | (0.6-2.1) | 25 | 5.5 | 1.3 | (0.6-2.7) |
| divorce | 10 | 0.2 | 1.0 | (0.5-2.1) | 5 | 0.3 | 0.8 | (0.3-2.2) | 7 | 0.6 | 1.4 | (0.5-3.6) | 10 | 1.6 | **3.5** | **(1.3-9.3)** | 5 | 1.1 | 3.1 | (0.8-11.4) |
| widow | 24 | 0.5 | 0.8 | (0.5-1.3) | 11 | 0.7 | 0.8 | (0.4-1.7) | 18 | 1.6 | 0.9 | (0.5-1.7) | 7 | 1.1 | 0.8 | (0.3-2.1) | 3 | 0.7 | 0.7 | (0.2-2.7) |
| Comorbidity | 96 | 2.1 | 1.0 | (0.8-1.4) | 78 | 5.0 | 1.0 | (0.7-1.6) | 89 | 8.0 | 1.1 | (0.8-1.7) | 75 | 11.7 | 1.0 | (0.5-2.0) | 44 | 9.7 | 1.4 | (0.6-3.2) |
| History of mental disorder | 126 | 2.7 | **4.4** | **(3.3-5.8)** | 73 | 4.7 | **4.8** | **(3.1-7.5)** | 125 | 11.2 | **4.6** | **(3.2-6.8)** | 71 | 11.1 | **6.1** | **(3.1-11.8)** | 40 | 8.8 | **3.5** | **(1.6-7.7)** |
| Chronic pain | 231 | 5.0 | **2.2** | **(1.5-3.2)** | 105 | 6.7 | **2.8** | **(1.5-4.9)** | 168 | 15.1 | **2.8** | **(1.7-4.8)** | 73 | 11.4 | **2.2** | **(1.2-4.3)** | 46 | 10.1 | **3.1** | **(1.3-7.1)** |
| Healthª, Mean±SD | 2.5 | 1.0 | **0.5** | **(0.4-0.6)** | 2.6 | 1.2 | **0.7** | **(0.5-0.8)** | 2.8 | 1.1 | **0.8** | **(0.7-1.0)** | 2.9 | 1.3 | 0.9 | (0.7-1.1) | 2.9 | 1.2 | 0.9 | (0.7-1.2) |
| SD, standard deviation; OR, odds ratio; CI, confidence interval; ªHigher score indicates a healthier status (range: 1-5); b Prevalence of severe psychological distress (K6≧13); c Adjusted for age, sex, education, employment, marital status, comorbidity, history of mental disorder, chronic pain, and health status | | | | | | | | | | | | | | | | | | | | |

| Appendix4 Odds ratio for severe psychological distress associated with restriction in healthcare utilization | | | | | | | | | | | | | | | | | | | | |
| --- | --- | --- | --- | --- | --- | --- | --- | --- | --- | --- | --- | --- | --- | --- | --- | --- | --- | --- | --- | --- |
|  | Hypertension | | | | Diabetes | | | | Respiratory disease | | | | Cardiovascular disease | | | | Cancer | | | |
|  | Nª | %ª | Adjusted OR ^b^ (95%CI) | | Nª | %ª | Adjusted OR ^b^ (95%CI) | | Nª | %ª | Adjusted OR ^b^ (95%CI) | | Nª | %ª | Adjusted OR ^b^ (95%CI) | | Nª | %ª | Adjusted OR ^b^ (95%CI) | |
| I got run out of medicines | 32 | 0.7 | **3.5** | **(2.1-5.7)** | 25 | 1.6 | **4.0** | **(2.1-7.7)** | 50 | 4.5 | **2.5** | **(1.5-4.1)** | 18 | 2.8 | 1.9 | (0.9-4.4) | 10 | 2.2 | 1.6 | (0.5-5.5) |
| My condition got deteriorated | 53 | 1.2 | **4.5** | **(2.9-7.1)** | 36 | 2.3 | **5.6** | **(3.1-10.0)** | 45 | 4.0 | **2.6** | **(1.6-4.3)** | 16 | 2.5 | 1.8 | (0.8-4.0) | 7 | 1.5 | 1.5 | (0.5-5.1) |
| I could not visit a hospital as scheduled or canceled a doctor visit | 84 | 1.8 | **2.7** | **(1.9-3.8)** | 41 | 2.6 | **2.4** | **(1.5-4.0)** | 72 | 6.5 | **1.9** | **(1.3-3.0)** | 27 | 4.2 | **2.2** | **(1.1-4.3)** | 10 | 2.2 | 1.2 | (0.4-3.4) |
| I could not visit a hospital for unexpected symptoms or ill condition | 50 | 1.1 | **3.0** | **(1.9-4.6)** | 31 | 2.0 | **4.3** | **(2.4-7.8)** | 50 | 4.5 | **1.8** | **(1.1-3.0)** | 18 | 2.8 | **2.2** | **(1.0-4.6)** | 6 | 1.3 | 1.0 | (0.3-3.6) |
| OR, odds ratio; CI, confidence interval; ª Prevalence of severe psychological distress (K6≧13); ^b^ Adjusted for age, sex, education, employment, marital status, comorbidity, history of mental disorder, chronic pain, and health status; ^c^ Adjusted for sex, education, employment, marital status, comorbidity, history of mental disorder, chronic pain, and health status; ^d^ Adjusted for sex, education, employment, marital status, comorbidity, history of mental disorder, and health status | | | | | | | | | | | | | | | | | | | | |
